# Marrow Adipose Tissue Expansion Coincides with Insulin Resistance in MAGP1-Deficient Mice

**DOI:** 10.3389/fendo.2016.00087

**Published:** 2016-06-30

**Authors:** Tezin A. Walji, Sarah E. Turecamo, Alejandro Coca Sanchez, Bryan A. Anthony, Grazia Abou-Ezzi, Erica L. Scheller, Daniel C. Link, Robert P. Mecham, Clarissa S. Craft

**Affiliations:** ^1^Department of Cell Biology and Physiology, Washington University School of Medicine, St. Louis, MO, USA; ^2^Department of Medicine and Medical Specialties, Faculty of Medicine and Health Sciences, University of Alcala de Henares, Madrid, Spain; ^3^Department of Medicine, Oncology Division, Washington University School of Medicine, St. Louis, MO, USA; ^4^Department of Medicine, Bone and Mineral Diseases Division, Washington University School of Medicine, St. Louis, MO, USA

**Keywords:** marrow adipose tissue, obesity, insulin resistance, bone remodeling, hematopoiesis, microfibril-associated glycoprotein-1

## Abstract

Marrow adipose tissue (MAT) is an endocrine organ with the potential to influence skeletal remodeling and hematopoiesis. Pathologic MAT expansion has been studied in the context of severe metabolic challenge, including caloric restriction, high fat diet feeding, and leptin deficiency. However, the rapid change in peripheral fat and glucose metabolism associated with these models impedes our ability to examine which metabolic parameters precede or coincide with MAT expansion. Microfibril-associated glycoprotein-1 (MAGP1) is a matricellular protein that influences cellular processes by tethering signaling molecules to extracellular matrix structures. MAGP1-deficient (*Mfap2*^−/−^) mice display a progressive excess adiposity phenotype, which precedes insulin resistance and occurs without changes in caloric intake or ambulation. *Mfap2*^−/−^ mice were, therefore, used as a model to associate parameters of metabolic disease, bone remodeling, and hematopoiesis with MAT expansion. Marrow adiposity was normal in *Mfap2*^−/−^ mice until 6 months of age; however, by 10 months, marrow fat volume had increased fivefold relative to wild-type control at the same age. Increased gonadal fat pad mass and hyperglycemia were detectable in *Mfap2*^−/−^ mice by 2 months, but peaked by 6 months. The development of insulin resistance coincided with MAT expansion. Longitudinal characterization of bone mass demonstrated a disconnection in MAT volume and bone volume. Specifically, *Mfap2*^−/−^ mice had reduced trabecular bone volume by 2 months, but this phenotype did not progress with age or MAT expansion. Interestingly, MAT expansion in the 10-month-old *Mfap2*^−/−^ mice was associated with modest alterations in basal hematopoiesis, including a shift from granulopoiesis to B lymphopoiesis. Together, these findings indicate MAT expansion is coincident with insulin resistance, but not excess peripheral adiposity or hyperglycemia in *Mfap2*^−/−^ mice; and substantial MAT accumulation does not necessitate a proportional decrease in either bone mass or bone marrow cellularity.

## Introduction

Adipose tissue exists in multiple variations, the most extensively studied being: brown, white, and beige. Brown adipocytes specialize in utilizing energy to produce heat (thermogenesis), white adipocytes are energy-storing reservoirs, and beige adipocytes principally store lipids but can be stimulated to transdifferentiate into a “brown-like” state (1). Less studied is a fourth lipid storing depot, located within the skeleton, marrow adipose tissue (MAT). These skeleton-associated adipocytes arise from a unique progenitor (2) and occupy approximately 70% of the marrow space within adult human bones (3). There is an evidence that MAT adipocytes exist in two forms: regulated marrow adipocytes (rMATs) that respond in size and number to physiological challenge, and the constitutive marrow adipocytes (cMATs) that persist despite challenge (4). Marrow adipocytes are not inert space-filling cells. Rather, the ability to produce leptin and adiponectin classifies them as an endocrine organ (5, 6). These cells also release fatty acids *via* lipolysis, suggesting that they may fuel local cellular processes (7).

Pathologic MAT expansion, as a consequence of metabolic dysfunction, has been studied in several contexts, including caloric restriction, high fat diet feeding, and leptin deficiency (6, 8–10). Unfortunately, the rapid change in peripheral fat and glucose metabolism associated with these models impedes our ability to determine which metabolic parameters precede or coincide with MAT expansion. The objective of this study was to correlate parameters of metabolic disease, bone remodeling, and hematopoiesis with MAT volume. Thus, we chose a model of progressive metabolic dysfunction – mice lacking the extracellular matrix (ECM) protein microfibril-associated glycoprotein-1 (MAGP1).

The ECM is a milieu of structural proteins, proteoglycans, and adhesive glycoproteins (11). MAGPs are a non-structural component of the ECM. The biology of this family has been recently reviewed (12). Briefly, MAGP1 (*Mfap2*) is broadly expressed, including both adipose tissue and bone (13, 14), and typically associates with fibrillin-rich microfibrils. MAGP1 may also interact with collagen-VI fibers (15). Characterization of MAGP1-deficient mice (*Mfap2*^−/−^) suggests that MAGP1 does not contribute to the mechanical integrity of its associated ECM fibers (16). Rather, MAGP1 functions as a gatekeeper of signal transduction. A significant body of evidence has demonstrated that MAGP1 interacts with ligands of the transforming growth factor beta (TGFβ) superfamily; sequestering them in the ECM (13, 14, 16, 17). In mice, MAGP1 deficiency (*Mfap2*^−/−^) results in adipocyte hypertrophy in peripheral white adipose tissue (WAT), diabetes, and reduced bone mass (13, 14). These phenotypes are progressive, developing with age.

Using the *Mfap2*^−/−^ model, we demonstrate that MAT expansion occurs concurrent with insulin resistance, not excessive peripheral adiposity or hyperglycemia. Bone loss in the MAGP1-deficient mice occurs without corresponding increases in MAT. Furthermore, a fivefold increase in MAT in the proximal tibia does not negatively affect bone marrow (BM) cellularity.

## Materials and Methods

### Animals and Diet

All animals were C57BL/6 background males, housed in a pathogen-free animal facility and fed standard chow *ad libitum*. MAGP1-deficient mice were generated using C57BL/6-derived ES cells [*Mfap2*^tm1a(KOMP)Wtsi^] purchased from KOMP Repository (Davis, CA, USA). ES cells were injected into blastocyst from C57BL/6 donors and transferred into pseudopregnant C57BL/6 females. Offspring were maintained on the Jackson Laboratories C57BL/6J strain. These mice are a newly derived line and are therefore different from the *Mfap2*^−/−^ mice used in previous studies (13, 14, 16, 17). All animals were treated in accordance with animal protocols approved by the Animal Studies Committee at Washington University.

### Tissue Collection

Tissues were harvested at 2, 6, and 10 months. Mouse fur was sprayed with 70% EtOH, then gonadal white adipose tissue (gWAT), and tibias were collected. Tissues were cleaned thoroughly of all contaminants (hair, connective tissue, etc.), then frozen or stored in 10% neutral buffered formalin.

### Insulin Tolerance Test

For insulin tolerance tests (ITTs), mice were fasted for 6 h and then given an injection of 0.75 U/kg Humulin-R insulin (Lilly, Indianapolis, IN, USA). Insulin was delivered by intraperitoneal injection, and tail blood glucose concentration was measured using Contour strips and meters (Bayer, Whippany, NJ, USA) at the indicated intervals.

### Osmium Staining and Micro-Computed Tomography

Micro-computed tomography (μCT) was completed on tibias from 2-, 6-, and 10-month-old mice. Bones were fixed in 10% neutral buffered formalin and then embedded in 2% agarose gel. Tibias were scanned at 20 μm voxel resolution using a Scanco μCT 40 (Scanco Medical AG, Zurich, Switzerland) calibrated using a hydroxyapatite phantom. Measurements of both trabecular and cortical bones were made based on reported guidelines (18). For trabecular bone, 50 slices below the growth plate were contoured to exclude the cortical bone, allowing trabecular bone volume/tissue volume (BV/TV) and bone mineral density (BMD) to be determined. For cortical bone, 20 slices – 2 mm proximal to the tibia–fibula junction were analyzed to determine cortical tissue mineral density (TMD). A threshold of 175 for trabecular bone and 260 for cortical bone (on a 0–1000 scale) was maintained. Note: two 10-month-old *Mfap2*^−/−^ bones were excluded from study due to suspected fracture.

Tibias were then decalcified in 14% EDTA for 3 weeks. Demineralized bones were incubated in a solution containing 1% osmium and 2.5% potassium dichromate for 48 h at room temperature. After thorough washing (water), tibias were embedded in 1% agarose gel. Osmium stained bones were then scanned as above, using a Scanco μCT 40, but at 10 μm voxel resolution. Analysis was performed in the same region as the trabecular bone (1,000 μm distal to the growth plate, 100 slices), using a threshold of 350 (on a 0–1000 scale). Osmium volume (OV) is quantification of the total OV within the region of interest. OV/TV is division of the OV by the volume of the marrow cavity within the same region.

### Flow Cytometry

Blood, spleen, and BM (one each of pelvis bone, femur, and tibia) were harvested from Jackson Laboratories c57BL/6J WT and *Mfap2*^−/−^ mice using standard techniques. Single cell suspensions were made, and red blood cells were lysed. Blood cells were counted using a Hemavet. BM and spleen cells were counted using a Cellometer. Ten million cells were stained and washed in FACS buffer (PBS, 1% BSA, 0.1% sodium aziade, and 2 μm EDTA). For monocyte, macrophage, and neutrophil analyses, cells from each tissue were stained with Brilliant Violet 421-conjugated F4/80 (BM8), peridinin chlorophyll protein complex (PerCP)-Cy5.5-conjugated MHC Class II (M5/114.15.2), phycoerythrin (PE)-Cy7-conjugated CD11c (N418), allophycocyanin (APC)-conjugated CD19 (1D3), APC-conjugated CD45R/B220 (RA3-6B2), APC-eFluor780-conjugated Ly-6G (Gr1, RB6-8C5), and PE-conjugated CD115 (AFS98). Two million live events were collected in the live gate and analyzed *via* FlowJo. Gating strategies can be found in Figure S1 in Supplementary Material.

### Statistical Analysis

**Figure 1:**
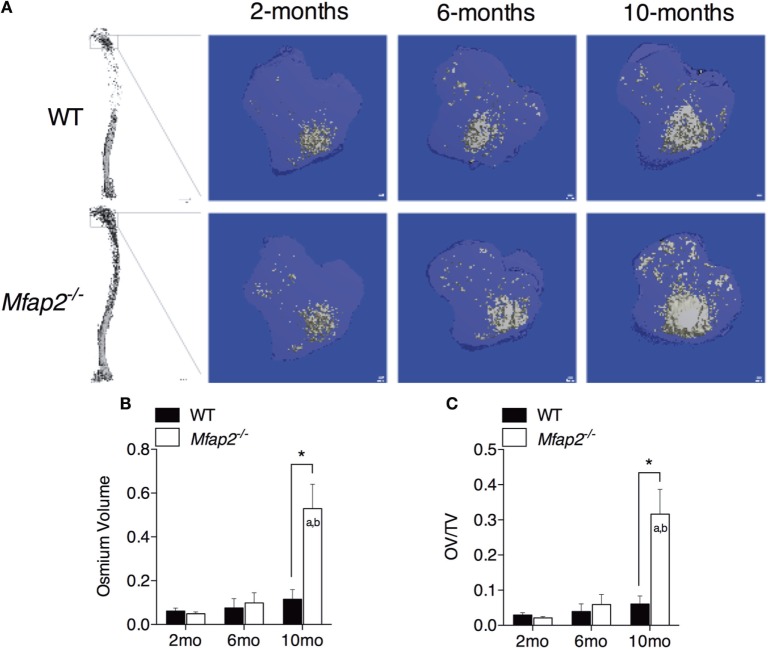
**MAT expansion in *Mfap2*^−/−^ mice**. **(A)** Micro-computed tomography (μCT) visualization of osmium stained lipid within the decalcified proximal tibia. **(B,C)** Region analyzed for osmium volume (OV) was 1 mm distal to the growth plate. OV/TV is osmium volume divided by the marrow cavity volume in the same region. Mean ± SEM; Sample sizes for WT and *Mfap2*^−/−^ mice, respectively: 2 months: *n* = 8, 8; 6 months: *n* = 6, 8; and 10 months: *n* = 8, 6. Statistical significance at each time point and within groups (WT or *Mfap2*^−/−^) over time was determined by two-way ANOVA with Holm–Sidak’s correction for multiple comparisons (*p ≤ 0.05, *a* = significant relative to 2 months of same genotype, and *b* = significant relative to 6 months of same genotype). Scale bar is 100 μm. Note: two 10-month-old *Mfap2*^−/−^ bones were excluded from study due to suspected fracture.

**Figure 2:**
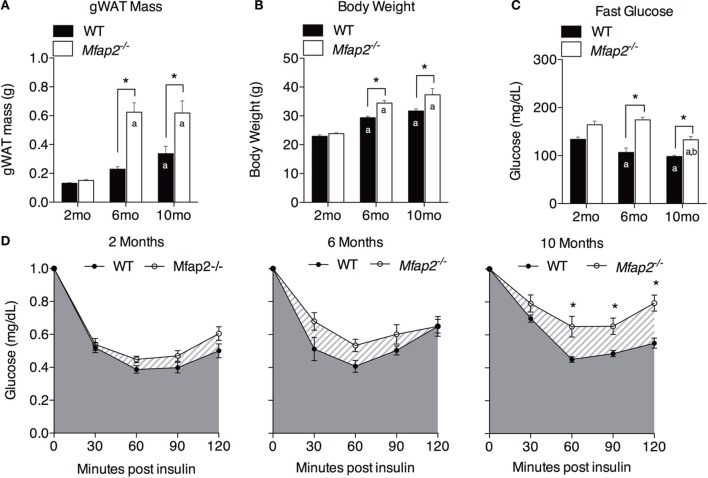
**Characterization of metabolic disorder in *Mfap2*^−/−^mice**. **(A)** Gonadal white adipose tissue (WAT) was harvested at 2 months (*n* = 10, 8), 6 months (*n* = 6, 8), and 10 months (*n* = 8, 8). **(B)** Total body weight at 2 months (*n* = 10, 8), 6 months (*n* = 7, 9), and 10 months (*n* = 8, 8). **(C)** Fasted (6 h) blood glucose concentration at 2 months (*n* = 10, 8), 6 months (*n* = 6, 8), and 10 months (*n* = 8, 8). **(D)** Insulin sensitivity results at 2 months (*n* = 10, 8), 6 months (*n* = 5, 8), and 10 months (*n* = 8, 8). Blood glucose data were normalized to time point 0 (before insulin injection). Mean ± SEM; statistical significance in **(A–C)** at each time point and within groups (WT or *Mfap2*^−/−^) over time was determined by two-way ANOVA with Holm–Sidak’s correction for multiple comparisons. Statistical significance in **(D)** was determined by repeated-measured two-way ANOVA with Sidak’s correction (*p ≤ 0.05, *a* = significant relative to 2 months of same genotype, and *b* = significant relative to 6 months of same genotype). Sample sizes shown above are for WT and *Mfap2*^−/−^ mice, respectively.

**Figure 3:**
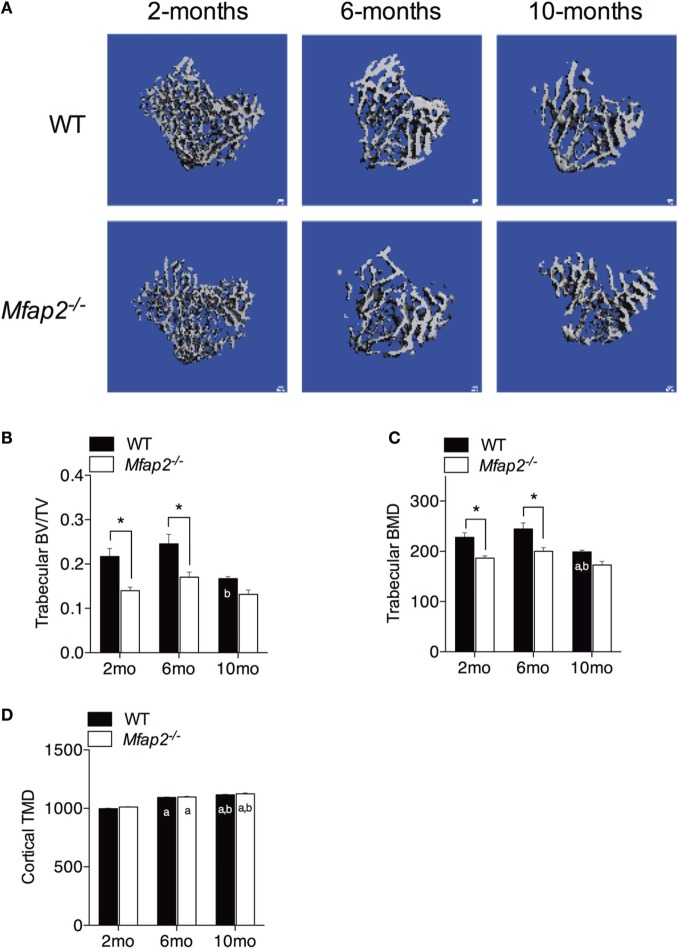
**Skeletal changes in *Mfap2*^−/−^ mice**. Tibias were harvested at 2, 6, and 10 months. Micro-comupted tomography was used to determine the ratio of bone volume to total volume (BV/TV) and bone mineral density (BMD/TMD). **(A–C)** For trabecular bone, 1 mm distal to the growth plate was analyzed. **(D)** For cortical bone, tissue mineral density (TMD) was determined by contouring 400 μm located 2 mm proximal to the tibia–fibula junction. Sample size for WT and *Mfap2*^−/−^ mice, respectively, are 2 months *n* = 8, 8; 6 months *n* = 6, 8; and 10 months *n* = 8, 6. Mean ± SEM; statistical significance at each time point and within groups (WT or *Mfap2*^−/−^) over time was determined by two-way ANOVA with Holm–Sidak’s correction for multiple comparisons (*p ≤ 0.05, *a* = significant relative to 2 months of same genotype, and *b* = significant relative to 6 months of same genotype). Scale bar is 100 μm. Note: two 10-month-old *Mfap2*^−/−^ bones were excluded from study due to suspected fracture.

**Figure 4:**
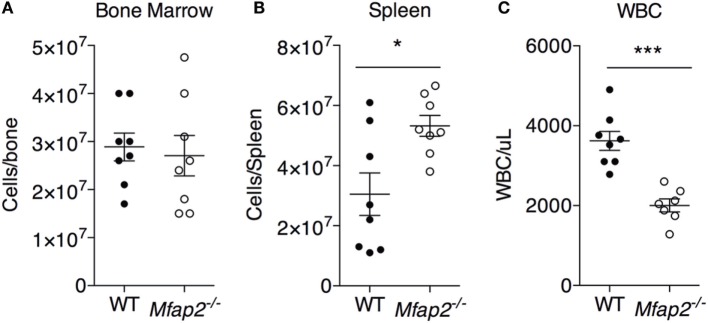
**MAT expansion and hematopoiesis**. To address the consequence of pathologic MAT expansion on hematopoiesis, the cellularity of the **(A)** bone marrow, **(B)** spleen, and **(C)** blood [white blood cells (WBCs)] were measured at 10 months. Sample size for WT and *Mfap2*^−/−^ mice, respectively, are *n* = 8, 8 **(A,B)** or *n* = 8, 7 **(C)**. Mean ± SEM; Student’s *t*-test was done for single comparison (*p ≤ 0.05 and ***p ≤ 0.001).

**Figure 5:**
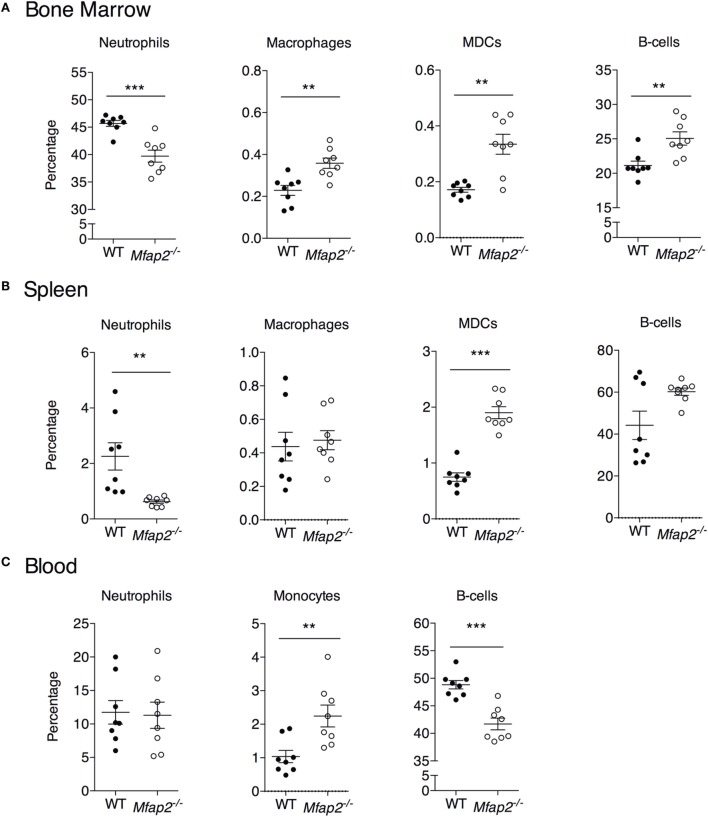
**Neutrophil, macrophage, myeloid-derived dendritic cell, monocyte, and B cell frequency within the (A) bone marrow, (B) spleen, and (C) blood at 10 months**. Neutrophils: B220^−^, Gr1^+^. Macrophage: B220^−^, Gr1^−^, MHCII^+^, F4/80^+^, and CD11c^−^. Dendritic cell: B220^−^, Gr1^−^, MHCII^+^, F4/80^−^, and CD11c^+^. Monocyte: B220^−^, Gr1^−^, and F4/80^+^. Sample sizes for WT and *Mfap2*^−/−^ mice, respectively, are *n* = 8 and 8. Mean ± SEM; Student’s *t*-test was done for single comparison (*p ≤ 0.05, **p ≤ 0.01, and ***p ≤ 0.001).

Statistical comparisons were performed using Graphpad Prism^®^ (GraphPad Software, Inc., La Jolla, CA, USA). In Figures 1–3, with the exception of panel 2D, comparisons between wild-type (WT) and *Mfap2*^−/−^ mice at each time point and over time within groups (WT or *Mfap2*^−/−^) were performed using a regular two-way ANOVA with Holm–Sidak correction for multiple comparisons. For the ITTs in panel 2D, repeated measures two-way ANOVA with Sidak’s correction was used. In Figures 4 and 5, results were compared with a two-tailed *t*-test. Comparisons with a *P*-value ≤0.05 were considered statistically significant.

## Results

### Pathologic MAT Expansion in *Mfap2*^−/−^ Mice

To compare parameters of metabolic disease with MAT expansion, a longitudinal study was performed in MAGP1-deficient (*Mfap2*^−/−^) mice that are susceptible to metabolic syndrome. Marrow adiposity was determined by μCT imaging of osmium stained lipid within the proximal tibia (Figure 1A). Quantification of total marrow lipid OV and OV normalized to total marrow cavity volume (OV/TV) is shown in Figures 1B,C. Marrow adiposity was found to be indistinguishable in either young (2 months) or adult (6 months) *Mfap2*^−/−^ mice, relative to WT mice. However, aged 10-month-old *Mfap2*^−/−^ mice displayed a fivefold increase in marrow lipid (OV and OV/TV) relative to WT animals. WT C57BL/6 mice have only a modest, statistically insignificant, increase (54%) in MAT between 6 and 10 months.

### Characterization of Metabolic Dysfunction in *Mfap2*^−/−^ Mice

Previously, we demonstrated that MAGP1 deficiency results in excess adiposity and reduced bone mass (13, 14, 16). Consistent with previous results, 2-month-old *Mfap2*^−/−^ mice had only a modest 15% increase in white fat (as measured by gonadal fat pad mass, Figure 2A). However, by 6 months, *Mfap2*^−/−^ mice had a 2.7-fold increase in fat mass, relative to WT mice. Extending our study to 10 months of age, we found that the differential in fat mass stabilizes, indicated by a 1.8-fold increase *Mfap2*^−/−^ gonadal fat pad mass relative to WT mice. Total body weight reinforced this finding (Figure 2B). *Mfap2*^−/−^ body weight was insignificantly increased at 2 months, but a significant (17%) increase in *Mfap2*^−/−^ weight was found at 6 months. Similar to the fat mass measurement, the weight differential between WT and *Mfap2*^−/−^ mice stabilized, remaining increased by 17% relative to baseline, at 10 months. Six-hour fasting blood glucose was significantly elevated in *Mfap2*^−/−^ mice by 2 months (12%, Figure 2C). By 6 months, fasted blood glucose was 63% higher in *Mfap2*^−/−^ mice relative to WT mice. However, by 10 months, there was only a 35% increase in *Mfap2*^−/−^ blood glucose relative to WT. To characterize metabolic function, ITTs were performed on WT and *Mfap2*^−/−^ mice at all ages (Figure 2D). In contrast to fat mass, body weight, and fasting blood glucose, insulin resistance did not reach statistical significance until the *Mfap2*^−/−^ mice were 10-month old. Thus, *Mfap2*^−/−^ mice are severely hyperglycemic by 6 months in age, but not insulin resistant until 10 months.

### Relationship between MAT Expansion and Bone Mass

Tibial bone morphology was assessed by μCT prior to osmium staining for MAT quantification (Figure 3A). Trabecular bone analysis was performed using the same region of interest analyzed for MAT (Figures 3B,C). Reduced trabecular bone volume (BV/TV) and BMD were detectable in *Mfap2*^−/−^ mice by 2 months. Aging of WT mice from 6 to 10 months was associated with statistically significant reductions in trabecular bone volume (−32%, Figure 3B) and mineral density (−19%, Figure 3C), but not a statistically significant increase in MAT (Figure 1C). During this time, *Mfap2*^−/−^ mice had a 22% reduction in BV/TV (Figure 3B), but a 5.2-fold increase in MAT volume (OV/TV, Figure 1C). Cortical bone density was maintained during aging and unchanged by MAT accumulation (Figure 3D). In fact, cortical BMD was highest at 10 months in both WT and *Mfap2*^−/−^ mice. Additional μCT indices of bone structure can be found in Tables S1 and S2 in Supplementary Material.

### MAT Expansion and Hematopoiesis

To address the impact of pathologic MAT expansion on hematopoietic function, we measured the cellularity of the BM and spleen as well as the number of circulating white blood cells (WBCs) at 10 months of age. BM cellularity was not significantly reduced in *Mfap2*^−/−^ mice, despite the fivefold increase in MAT (Figure 4A). However, spleen cellularity was slightly elevated and WBC count was decreased in *Mfap2*^−/−^ mice (Figures 4B,C). To further investigate these findings, flow cytometry was used to assess hematopoietic lineage distribution. Within the BM of *Mfap2*^−/−^ mice, there was a modest increase in macrophages, myeloid-derived dendritic cells (MDCs), and B-cells (+61, +78, and +18%, respectively) and a corresponding decrease in neutrophils relative to WT mice (−13%) (Figure 5A). In the spleen, there was a significant increase in MDCs (+250%) and decrease in neutrophils (−72%), with a trend to increased B-cells (Figure 5B). Finally, in the blood, we observed an increase in monocytes (+216%) and a modest decrease in B-cells (−15%) in *Mfap2*^−/−^ mice (Figure 5C).

## Discussion

Once viewed as inert, space-filling structures, BM adipocytes are now recognized as metabolically active cells with the potential to influence bone remodeling and hematopoiesis. The relationship between MAT volume and peripheral fat mass has been addressed previously (9, 10). In rodents, weight gain resulting from HFD feeding is associated with significant MAT expansion. However, extreme peripheral fat loss due to caloric restriction also correlates with increased marrow adiposity (6). The rapid change in adiposity impedes our understanding of whether changes in peripheral adipose tissue lipid storage shift lipids to the marrow, or if metabolic changes such as insulin resistance contribute to MAT expansion. To address this question, we performed a longitudinal study of mice predisposed to excess, age-associated fat accumulation without dietary intervention.

Microfibril-associated glycoprotein-1 is a matricellular protein that associates with ECM structures but does not contribute to their biophysical properties. Instead, MAGP1 affords fibrillar structures the capacity to regulate signal transduction by tethering growth factors to the ECM [reviewed in Ref. (12)]. MAGP1 is expressed in adipose tissue. MAGP1 deficiency in mice (*Mfap2*^−/−^) causes adipocyte hypertrophy in peripheral WAT, which contributes to obesity and then insulin resistance (13). MAGP1 is also highly expressed by osteoblasts in the bone. Loss of MAGP1 *in vivo* leads to increased osteoclast number and reduced bone mass (14). In this study, MAT expansion did not occur coincident with bone loss or excess peripheral adiposity in *Mfap2*^−/−^ mice; osteopenia was detectable by 2 months and excess adiposity by 6 months in *Mfap2*^−/−^ mice whereas pathologic MAT expansion was not detectable until 10 months of age. This suggests that MAGP1 deficiency in bone is sufficient to cause abnormal bone remodeling but is unlikely to be mediating the changes in MAT. Instead, MAT expansion coincided with insulin resistance in *Mfap2*^−/−^ mice. Our findings are of importance because they address three confounding factors of MAT biology: the mechanisms that drive MAT expansion, the relationship between MAT expansion and bone loss, and whether MAT is detrimental to hematopoiesis.

The coincidence of MAT expansion with insulin resistance suggests that insulin may limit MAT adipocyte size or number. Insulin signaling in marrow adipocytes has not been reported, thus, we cannot comment on insulin’s direct action on lipid storage or adipogenesis in these specialized cells. However, the pathophysiology of insulin resistance could induce a secondary complication (e.g., hyperlipidemia) that drives subsequent MAT expansion. Insulin is a potent inhibitor of lipolysis, the breakdown of triglyceride stores, in traditional white adipocytes. Insulin resistance is therefore associated with elevated circulating free fatty acids (FFA) and excess energy storage in atypical sites (e.g., muscle and liver) (19). It is possible that marrow adipocytes respond to elevated FFA associated with insulin resistance by storing them as triglycerides. Rodent models of type 1 diabetes (T1DM) also display significant MAT accumulation (20–22), providing a second example of perturbed insulin signaling associating with hyperlipidemia and MAT expansion.

The relationship between marrow adiposity and bone mass is highly debated. Published reports have shown that MAT expansion can coincide with increased, reduced, or unchanged bone volume (9, 10, 23–27).

Substantial *in vitro* evidence demonstrates that adipocytes and osteoblasts arise from the same mesenchymal progenitor. If true, maturation of mesenchymal stem cells through the adipocyte lineage should be at the expense of the osteoblast lineage. However, *in vivo* evidence is conflicting. Specifically, lineage tracing of marrow adipocytes has demonstrated that the osteoblasts are derived from a Gremlin-1 positive mesenchymal progenitor cell, but these Gremlin-1 positive cells do not differentiate into adipocytes (28). This would allow osteoblast cell numbers to exist independent of marrow adipocyte number. The marrow cavity is also a relatively defined space, and it would, therefore, seem that expansion of MAT must occur at the expense of something else, such as bone. Our study supports a disconnection between MAT volume and bone volume. When WT and *Mfap2*^−/−^ mice age from 6 to 10 months, there is an increase in MAT and decrease in BV/TV. However, loss of trabecular BV/TV from 6 to 10 months is actually greater in WT mice, despite *Mfap2*^−/−^ mice having a greater accrual of MAT. Specifically, in WT mice, BV/TV loss is 32% and MAT volume increases 54%; however, MAT volume in *Mfap2*^−/−^ mice is increased 530% but BV/TV is decreased only 22%.

Marrow adipocytes are considered as members of the hematopoietic niche. Marrow adiposity and hematopoiesis have been studied in the context of marrow ablation by irradiation and chemotherapy. From these studies, it was concluded that the filling of the marrow with fat impedes marrow reconstitution and hematopoiesis (29–31). The current study investigated the consequence of pathologic MAT expansion on existing hematopoietic cells (not reconstitution). We found a significant reduction in circulating WBCs and a small increase in spleen cellularity, indicators of BM failure and extramedullary hematopoiesis. Further study is needed to determine whether MAT expansion in this model leads to a loss of functional hematopoietic stem cells. Unexpectedly, however, total BM cellularity was unchanged. This may be due to shifts in marrow composition to favor loss of fluid space and vascular tone with MAT expansion to allow subsequent preservation of hematopoiesis. We also examined whether fat expansion within the marrow would cause a recruitment (or retention) of macrophages. Macrophages are recruited to peripheral adipose tissue to allow remodeling of adipose tissue during expansion, and because of this, obesity is linked to fat inflammation (32). Indeed, we found a slight increase in macrophage frequency within *Mfap2*^−/−^ BM. However, the implications of this finding are unclear. Future studies are needed to examine the remodeling of the MAT adipocyte niche in response to obesity.

The goal of this project was to determine the association between pathologic MAT expansion and peripheral indicators of metabolic dysfunction without the use of modified diet. We also addressed the relationship between MAT expansion and bone mass, and the consequence of pathologic MAT expansion on hematopoiesis. We found that MAT volume was not linked directly to peripheral fat accumulation and, instead, coincided with insulin resistance. Furthermore, we found that bone loss occurred, regardless of MAT expansion, in *Mfap2*^−/−^ relative to WT at all ages. Lastly, we show that MAT expansion is associated with modest alterations in basal hematopoiesis, including a shift from granulopoiesis to B lymphopoiesis.

## Author Contributions

TW and ST contributed equally to this work. TW, ST, AS, and BA designed and performed the experiments as well as analyzed the data. TW, ST, and CC wrote the manuscript. GA-E and DL analyzed and interpreted FACS data. CC, ES, RM, and DL provided research materials and contributed to editing of the manuscript.

## Conflict of Interest Statement

The authors declare that the research was conducted in the absence of any commercial or financial relationships that could be construed as a potential conflict of interest.

## Abbreviations

ECM, extracellular matrix; MAT, marrow adipose tissue; MAGP1, microfibril-associated glycoprotein-1.
