# Supplementary material for: Marrow Adipose Tissue Expansion Coincides with Insulin Resistance in MAGP1-Deficient Mice
Source: Front Endocrinol (Lausanne). 2016 Jun 30;7:87. doi: 10.3389/fendo.2016.00087 (PMC4928449; doi:10.3389/fendo.2016.00087)
Supplement: Supplementary file 1 [file Table_1.DOC]

**Supplemental Table 1: Microcomputed tomography analysis of WT and *Mfap2^-/-^* trabecular bone**

**2-months 6-months 10-months** Genotype Mean P-value Genotype Mean P-value Genotype Mean P-value **VOX-TV (mm^3^)**WT

*Mfap2-/­-*2.10

2.30 0.092WT

*Mfap2-/­-*1.92

1.73 **0.002**WT

*Mfap2-/­-*1.83

1.70 **0.040 VOX-BV (mm^3^)**WT

*Mfap2-/­-*0.449

0.322 **0.004**WT

*Mfap2-/­-*0.468

0.295 **0.0004**WT

*Mfap2-/­-*0.307

0.225 **0.004 VOX-BV/TV** WT

*Mfap2-/­-*0.216

0.139 **0.002**WT

*Mfap2-/­-*0.245

0.170 **0.006**WT

*Mfap2-/­-*0.167

0.131**0.004 Connectivity Density** WT

*Mfap2-/­-*155.25

84.13 **0.001**WT

*Mfap2-/­-*79.40

52.14**0.044**WT

*Mfap2-/­-*33.03

25.43 0.186 **Structure Model Index** WT

*Mfap2-/­-*2.60

3.20 **0.003**WT

*Mfap2-/­-*1.87

2.41 **0.046**WT

*Mfap2-/­-*2.09

2.63 **0.006 Trabecular Number** WT

*Mfap2-/­-*6.23

5.51 **0.0007**WT

*Mfap2-/­-*5.11

4.56 **0.050**WT

*Mfap2-/­-*3.65

3.83 0.348 **Trabecular Thickness (mm)**WT

*Mfap2-/­-*0.056

0.049 **0.005**WT

*Mfap2-/­-*0.070

0.062 **0.001**WT

*Mfap2-/­-*0.071

0.065**0.004Trabecular Spacing (mm)**WT

*Mfap2-/­-*0.156

0.180 **0.001**WT

*Mfap2-/­-*0.189

0.215 0.053WT

*Mfap2-/­-*0.276

0.263 0.400**Bone Mineral Density**

**(mg HA/ccm)**WT

*Mfap2-/-­*227.96

186.54 **0.0008**WT

*Mfap2-/­-*244.45

200.10 **0.005**WT

*Mfap2-/­-*198.86

172.78**0.003**
