# Supplementary material for: Marrow Adipose Tissue Expansion Coincides with Insulin Resistance in MAGP1-Deficient Mice
Source: Front Endocrinol (Lausanne). 2016 Jun 30;7:87. doi: 10.3389/fendo.2016.00087 (PMC4928449; doi:10.3389/fendo.2016.00087)
Supplement: Supplementary file 2 [file Table_2.DOC]

**Supplemental Table 2: Microcomputed tomography analysis of WT and *Mfap2^-/-^* cortical bone**

**2-months 6-months 10-months** Genotype Mean P-value Genotype Mean P-value Genotype Mean P-value **DT-Ct.Th (mm)**WT

*Mfap2-/­-*0.187

0.175 **0.029** WT

*Mfap2-/­-*0.204

0.197 **0.014** WT

*Mfap2-/­-*0.205

0.192 **0.007 TMD**

**(mg HA/ccm)**WT

*Mfap2-/­-*997.30 1012.29 **0.012** WT

*Mfap2-/­-*1093.73 1098.33 0.559 WT

*Mfap2-/­-*1117.06 1129.66 0.121 **Total Area (mm^2^)**WT

*Mfap2-/­-*1.14

1.06 **0.005** WT

*Mfap2-/­-*1.3

1.1 **3.83 E-05** WT

*Mfap2-/­-*1.3

1.2 0.108 **Bone Area**

**(mm^2^)**WT

*Mfap2-/­-*0.609

0.543 **0.004** WT

*Mfap2-/­-*0.73

0.64 **0.0003** WT

*Mfap2-/­-*0.73

0.67 **0.018 Medullary Area (mm^2^)**WT

*Mfap2-/­-*0.539

0.522 0.191 WT

*Mfap2-/­-*0.55

0.46 **9.42 E-05** WT

*Mfap2-/­-*0.63

0.61 0.548 **pMOI [mm^4]** WT

*Mfap2-/­-*0.168

0.130 **0.012** WT

*Mfap2-/­-*0.205

0.154 **6.75 E-05** WT

*Mfap2-/­-*0.222

0.202 0.202
