# Supplementary material for: Marrow Adipose Tissue Expansion Coincides with Insulin Resistance in MAGP1-Deficient Mice
Source: Front Endocrinol (Lausanne). 2016 Jun 30;7:87. doi: 10.3389/fendo.2016.00087 (PMC4928449; doi:10.3389/fendo.2016.00087)
Supplement: Supplementary file 3 [file Data_Sheet_1.PDF]

Suplemental Figure 1:

A. Bone Marrow

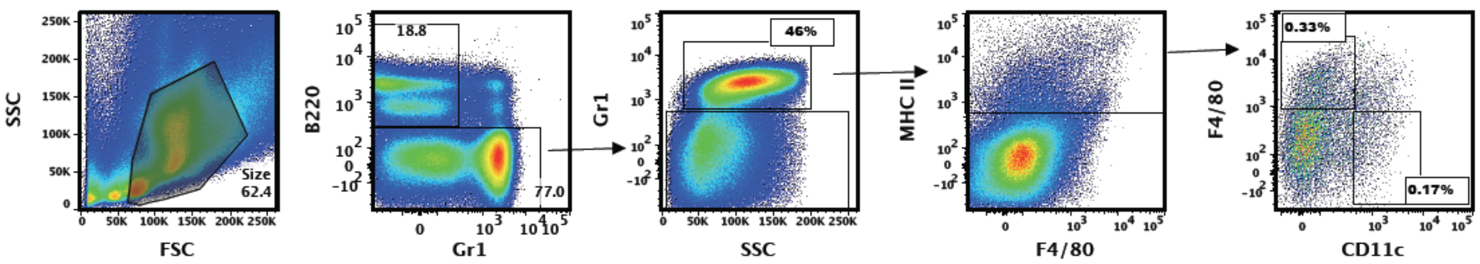

B. Spleen

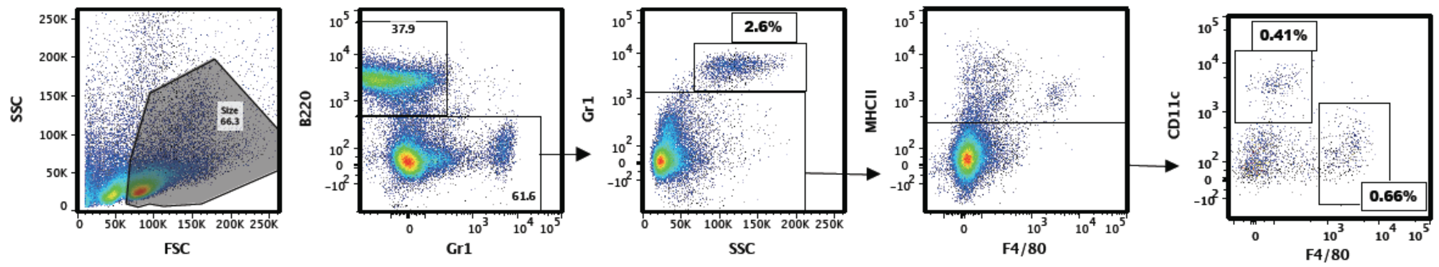

C. Blood

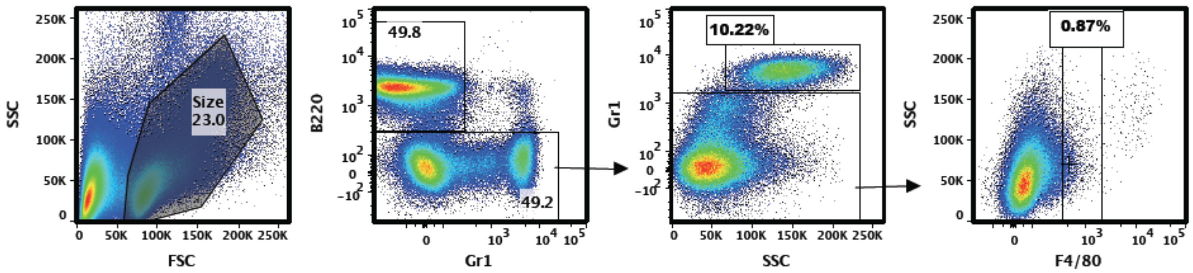

S1: Representative gating strategy for FACS analysis of bone marrow (A), spleen (B), and blood (C) samples.
